# Effect of gallium maltolate on a model of chronic, infected equine distal limb wounds

**DOI:** 10.1371/journal.pone.0235006

**Published:** 2020-06-19

**Authors:** Shauna P. Lawless, Noah D. Cohen, Sara D. Lawhon, Ana M. Chamoun-Emanuelli, Jing Wu, Andrés Rivera-Vélez, Brad R. Weeks, Canaan M. Whitfield-Cargile

**Affiliations:** 1 Department of Large Animal Clinical Sciences, College of Veterinary Medicine & Biomedical Sciences, Texas A&M University, College Station, Texas, United States of America; 2 Department of Veterinary Pathobiology, College of Veterinary Medicine & Biomedical Sciences, Texas A&M University, College Station, Texas, United States of America; 3 Veterinary Medical Teaching Hospital, College of Veterinary Medicine & Biomedical Sciences, Texas A&M University, College Station, Texas, United States of America; Western College of Veterinary Medicine, University of Saskatchewan, CANADA

## Abstract

Distal limb wounds are common injuries sustained by horses and their healing is fraught with complications due to equine anatomy, prevalence of infection, and challenges associated with wound management. Gallium is a semi-metallic element that has been shown to possess antimicrobial properties and aid in wound healing in various preclinical models. The effects of Gallium have not been studied in equine wound healing. Therefore, the objective of this study was to compare healing rates between gallium-treated and untreated wounds of equine distal limbs and to demonstrate the antimicrobial effects of gallium on wounds inoculated with *S*. *aureus*. Using an established model of equine wound healing we demonstrated beneficial effects of 0.5% topical gallium maltolate on equine wound healing. Specifically we documented reduced healing times, reduced bioburden, and reduced formation of exuberant granulation tissue in wounds treated with gallium maltolate as compared with untreated wounds. Gallium appeared to exert its beneficial effects via its well-described antimicrobial actions as well as by altering the expression of specific genes known to be involved in wound healing of horses and other animals. Specifically, gallium maltolate appeared to increase expression of transforming growth factor-β in both infected and un-infected wounds. Further work is needed to document the effects of gallium on naturally occurring equine wounds and to compare the effects of gallium with other wound treatment options. These data, however, suggest that gallium may be an attractive and novel means of improving equine distal limb wound healing.

## Introduction

Equine distal limb wounds are among the most common injuries evaluated by equine practitioners. Healing of these wounds is challenging because of factors such as vascular supply and motion. Healing is further complicated by the prevalence of bacterial infections and difficulty in suturing wounds closed due to loss of soft tissue. [1–3] In fact, only 26% of wounds are able to be successfully closed to heal by primary intention; open wounds often become infected, which contributes to complications such as delayed healing, osseus sequestra, excessive granulation tissue, restrictive scar tissue formation, and infection of the surrounding tissues. [2–10] *Staphylococcus* spp. and *Pseudomonas* spp. are the most common bacterial genera isolated from equine wounds, and *Staphylococcus aureus (S*. *aureus)* is among the most common species isolated. [11, 12] In addition, it is estimated that 5% to 12% of healthy horses are colonized with methicillin-resistant *S*. *aureus* (MRSA), but it is unknown how frequently naturally occurring wounds become infected with MRSA. [13, 14] Given the increased prevalence of MRSA and other drug-resistant bacteria, and the ease of transfer of such organisms between horses and people, the prevalence of equine wounds infected with multidrug-resistant bacteria can be expected to rise.

Gallium is a semi-metallic element whose chemical properties closely resemble those of ferric iron (Fe^3+^). This results in Ga^3+^ being taken up by bacteria to be used in biological processes requiring Fe^3+^, including DNA synthesis, resulting in inhibition of bacterial replication and ultimately bacterial death. [15] Gallium maltolate (GaM) has been demonstrated to be effective against *Staphyloccocus* spp (including MRSA), *Rhodococcus equi*, *Pseudomonas aeruginosa*, *Mycobacterium avium* subspecies *paratuberculosis*, *Porphyromonas ginigvalis*, *Francisella tularensis*, and multidrug-resistant *Acinetobacter baumannii*. [16–26] An additional property of gallium is its ability to decrease formation of biofilms, a major cause of delayed healing reported to occur in 62% of equine wounds. [11, 12, 22] Additionally, gallium can promote effective wound healing through several anti-inflammatory and anti-proliferative properties by modulating the chronic inflammatory response and reducing unwanted proliferation of fibroblasts (as occurs with proliferative granulation tissue, or “proud flesh” formation). [25–29] These combined antimicrobial, anti-inflammatory, anti-biofilm, and anti-proliferative properties of gallium make it an attractive therapy to aid in the healing of equine wounds. To the authors’ knowledge, there have been no studies examining the effects of gallium on equine would healing and the only information about GaM on equine wound healing comes from anecdotal reports of a commercially-available, non-FDA approved product currently being used by some equine practitioners and horse-owners in North America. Therefore, the objective of this study was to compare healing rates between GaM-treated and untreated wounds of equine distal limbs and to demonstrate the antimicrobial effects of GaM on wounds inoculated with *S*. *aureus*. Based upon studies in other animal species, known biological effects of gallium, and known disturbances in equine wound healing we hypothesized that: 1) equine wounds treated with GaM will reduce in size significantly more quickly, form significantly less exuberant granulation tissue, and have significantly improved microscopic pathology scores (based on inflammation, epithelialization, angiogenesis, and fibroplasias) than wounds treated without GaM; and, 2) GaM treatment will significantly decrease the bioburden of wounds.

## Materials and methods

### Study population

Horses (n = 6) donated to Texas A&M College of Veterinary & Biomedical Sciences for euthanasia for reasons unrelated to dermatologic conditions and with no evidence or history of skin wounds on the distal limbs were utilized for this project. Horses donated for this project consisted of 2 mares and 4 geldings (2 American Quarter horses, 1 draft cross, 1 appendix, 1 paint horse, and 1 horse of unknown breed. The mean age was 7 years (4–12). The mean weight was 446 kg (386–545). One horse was used for non-infected wound model and 5 horses were used for the infected wound model. Horses were confined to a 3.7 x 3.7 meter stall with other horses in adjacent stalls and with natural light. The protocol for this study was approved by the Texas A&M University Institutional Animal Care and Use Committee (IACUC 2013–0264).

### Non-infected wounds

The surgical model used to induce exuberant granulation tissue in equine wounds has been well-characterized. [30–33] Anesthesia was induced and maintained as previously described. [34] Briefly, horses were sedated with xylazine (Anased, 1.1mg/kg IV) and ketamine (Ketaject, 3mg/kg IV) and maintained on a constant rate infusion (CRI) of 5% guafensin with 1,000 mg ketamine and 500 mg xylazine per liter (GKX) administered at a rate of 2mL/kg/hour. Horses were placed in dorsal recumbency. The distal forelimbs from the level of the distal carpus to the distal fetlock joint were clipped circumferentially and aseptically prepared. Beginning 2 cm proximal to the fetlock, a 2.5 cm x 2.5 cm full-thickness skin incision was made using a sterile template, and the resulting 6.25 cm^2^ section of skin was removed using a combination of sharp and blunt dissection. This was repeated 4 times on the lateral aspect of each forelimb so that a vertical column of 2.5 cm x 2.5 cm wounds were created, separated by 2 cm of normal intact skin (**S1 Fig**). The skin removed at the time of surgery was preserved (as described later) and used as normal skin to compare gene expression data between treated and untreated wounds. A sterile distal limb bandage was then applied to both forelimbs, in a manner previously described. [30] The distal limb bandages were changed twice weekly throughout the study period. The granulation tissue beds on each limb were given 10 days to mature. At day 10, treatment and placebo treatment was initiated. GaM 0.5% in a petroleum base (Aquaphor® Beiersdorf Inc) was applied to all 4 wounds of 1 limb, while all 4 wounds on the contralateral (control) limb were treated with drug-vehicle petroleum ointment. Limb treatment was assigned randomly based on a coin toss. GaM 0.5% (1.25 mL per wound) and petroleum vehicle (1.25 ml per wound) were applied to the treatment and control wounds, respectively, with each bandage change (twice weekly) for the duration of the study period. Each of the 4 wounds on each leg were biopsied at day 21 of treatment. Biopsies were performed under standing sedation with detomidine (0.01 mg/kg IV) and butorphanol (0.01 mg/kg), and a ring-block around the proximal metacarpus using 240 mg of 2% mepivicaine subcutaneously. An 8-mm punch biopsy was used to obtain a biopsy specimen from each wound centred on the wound margin, such that each biopsy contained granulation bed, wound margin, and a small portion of normal surrounding tissue. Each wound was digitally photographed once weekly (at the time of every other bandage change) prior to application of treatment such that images were acquired on days 0 (time 1), 7 (time 2), 14 (time 3), and 21 (time 4). Wound size was determined digitally using image analysis software (Image J ver. 1.51). [35, 36] Granulation tissue height was determined at the same time-points with a contour gauge (**S2 Fig**). Gene expression analysis was performed as described later.

### Infected wounds

Wounds were created, as described, for non-infected wounds and granulation tissue allowed to develop for 10 days. All 4 wounds on both legs were inoculated with *S*. *aureus*, by placing a cotton gauze the same size as the surface area of the wound, inoculated with 5 × 10^7^ colony forming units (CFUs), directly on the surface of the wound and then bandaging in place, as described previously. [37] The inoculated gauze was left in place for 48 hours as this is the amount of time reported for the wounds to become infected and a biofilm to form. [38] After 48 hours of contact with *S*. *aureus* inoculated gauze (time 1), the gauze was removed and treatment was applied as described for non-infected wounds. Control and treated wounds were biopsied weekly (time 2 = 7 days, time 3 = 14 days, time 4 = 21 days) using the technique described above to obtain longitudinal data. Horses were euthanized at the end of the study. Euthanasia was accomplished by induction of general anaesthesia as described above followed by intravenous injection of an overdose of pentobarbital containing euthanasia solution (Vet One Euthanasia Solution, Boise, Idaho).

### Wound size and granulation height

Wound size was captured from digital photographs and wound area was measured in triplicate, by an observer blinded to treatment group, and the mean of three measurements of wound area calculated. [35, 36] Similarly, granulation tissue height, acquired from the contour gauge, was measured in triplicate. The maximum height of each measurement was recorded and the average of 3 measurements was calculated (**S2 Fig**).

### Microscopic pathology

Each biopsy sample was processed aseptically and divided into 3 equal-sized, tranverse sections. The middle 1/3 of the biopsy specimen was formalin fixed, paraffin embedded, stained using H&E and then scored semi-quantitatively using a 0 to 3 scoring system (based on inflammation, epithelialization, angiogenesis and fibroplasia) modified from a previously described system. [32] Briefly, the previous scale was transposed such that a lower score represented improved healing and a higher score represented poor healing. Specifically, inflammation was scored 0 (absent) to 3 (diameter of inflammatory focus measured more than 5 times the intact epidermis), epithelialization was scored 0 (complete epithelialization) to 3 (no new epithelium at wound margin, angiogenesis was scored 0 (more than 15 capillaries present in the wound bed to 3 (no new capillaries present), and fibroplasia was scored 0 (no fibroblast apparent) to 3 (well-aligned fibroblast with their long axes parallel to one another and to the wound surface. The cumulative scores were then summed to create a single microscopic pathology score. Microscopic pathology was performed by a board-certified veterinary pathologist blinded to treatment group.

#### Bacterial quantification

One remaining sample was utilized for quantitative culture as previously described. [39] Briefly, the sample was weighed, placed in 5 ml sterile phosphate buffered saline (PBS—VWR Life Science, Sanborn, NY), and homogenized for 90 seconds at 6.5 m/sec with FastPrep FP120 cell disrupter (Qbiogene, Carlsbad,CA). *S*. *aureus* CFU were quantified by plating 10-fold serial dilutions on CHROMagar^TM^ *S*. *aureus* selective plates (CHROMagar Microbiology, Paris, France) and counting colonies of *S*. *aureus* after 24 hours of incubation at 37°C in order to calculate CFU/gram of tissue. [40, 41] In addition to quantitative culture, concentration of *S*. *aureus* was also quantified via quantitative real time PCR (qPCR). Briefly, DNA was isolated from the remaining homogenate using a commercially available kit (QIAamp DNAStool Mini Kit, Qiagen, Düsseldorf, Germany) according to manufacturer’s protocol. [42] Detection of *S*. *aureus* was performed via qPCR as previously described. [43, 44] Briefly, the *S*. *aureus*-specific gene sa442 was amplified using the forward primer 5’- CAATCTTTGTCGGTACACGATATTCT-3’, reverse primer 5’- CAACGTAATGAGATTTCAGTAGATAATACAAC-3’, and a combination of two probes 5’- CACGACTAAATAAACGCTCATTCGCGATTTT-3’and 5’- CACGACTAAATAGACGCTCATTCGCAATTTT-3’ each labelled with FAM at the 5’end and black hole quencher 1 at the 3’end. [44] Samples were run in triplicate in a 25μL reaction volume using Taqman Fast Universal PCR Master Mix (Cat. 4352046, ThermoFisher Scientific; Waltham, MA, USA) with each primer used at 500 nM final concentration and the probe mixture at a final concentration of 500 nM. A standard curve was included in every run to allow quantification of the number of bacteria present in the original sample. Each standard curve consisted of serial 10-fold dilution series of DNA from *S*. *aureus* (ATCC29213) as a positive control and a negative control consisting of *S*. *pseudintermedius* DNA from a clinical isolate as well as a negative control without template DNA added to the reaction mixture.

### Host gene expression

The remaining 1/3 of the original biopsy specimen was used to quantify host gene expression. Briefly, the sample was submersed in 500 μL of AllProtect^®^ Tissue Reagent (Qiagen, Düsseldorf, Germany) immediately following collection, refrigerated at 4°C for 24 hours, then frozen at -20°C for analysis at a later date. These samples were then thawed on ice and mRNA extracted using a commercially available kit (RNeasy Mini kit, Qiagen, Düsseldorf, Germany) according to manufacturer’s protocol. Gene expression levels were measured via Reverse transcription-qPCR (RT-qPCR) (qScript One-Step Kit, Quanta Biosciences, Gaithersburg, MD) using the primers shown in Table 1. These primers were developed in-house using PrimerQuest® program, IDT, Coralville, Iowa, USA. Accessed Feburary, 2017. https://www.idtdna.com/SciTools. Gene expression was normalized to GAPDH and data are expressed as mean (95% CI) relative to normal skin.

**Table 1:** Genes and primers used for RT-qPCR to determine host gene expression.

Gene	Primer	Sequence (5`- 3`)
*IL-10*	Sense	GCA GGG TGA AGA CTT TCT TTC AAA TG
	Antisense	CACTCATGGCTTTGTAGACACCTTTC
*IL-1β*	Sense	CCAAAACAGATGAAGGGCAGC
	Antisense	TGA GCA GGG AAC GGG TAT CT
*IL-2*	Sense	CCC AAG AAG GCC ACA GAA TTG A
	Antisense	TGT TTC AGA CCC CTT TAG TCC CA
*IFN-γ*	Sense	GTG CGA TTT TGG GTT CTT CTA CC
	Antisense	TCA CTA TCC TCT TTC CAG TTC TTC AAG
*CXCL1*	Sense	CCCAAACCGAAGTCATAGCCA
	Antisense	GCC AAC AGC AAC CAG TAC ACT
*PDGF-A*	Sense	CCC GTC ATC ATC TTG GGG AA
	Antisense	GGA TGG CCT CCT CGA TGC
*VEGF-A*	Sense	ATCTTCCAGGAGTACCCCGA
	Antisense	GGTGAGGTTTGATCCGCATAATCTG
*TGF-β*	Sense	GCTACCTCAGTAACCGGCTG
	Antisense	CTG GAA CTG AAC CCG TTG ATG C
*MMP1*	Sense	GCTGAG TTTGTCCTCA CCGAA
	Antisense	GTC ACG ATG ATC TCC CCT GAC A
*MMP7*	Sense	GTG GAA ACA GGC TCA GGA CTA TC
	Antisense	TCTGCAACATCTGGCACTCC
*MMP9*	Sense	CCT GCC ACT TCC CCT TCA C
	Antisense	CCGTCCTGGGTGTAGAGTTTCTC
Serpine	Sense	GTGGAACAAGGACGAGATCACCA
	Antisense	GTC GCT GAT CAT GCC TTT TGT GT
*TIMP1*	Sense	AACCAGACCACCTTACAGCG
	Antisense	GTCCAATAGTTGTCCGGCGA
*COL1A1*	Sense	GGTGCTGATGGCCAACCT
	Antisense	ACC AGT AGC ACC AGG GGG A
*COL14A1*	Sense	AACTGTTTGCCATAGGGGTGAA
	Antisense	GGCATGAGTCCAGTTCACCA
Vitronectin	Sense	CGGGAGCGGGTCTACTTTTT
	Antisense	GCTGTCCAGTACCACCAGAG
*GAPDH*	Sense	CGCCATCAATGACCCCTTCA
	Antisense	GGATCTCGCTCCTGGAAGATG

Primers used to quantify host gene expression via RT-qPCR

### Data analysis

Data were analyzed using linear-mixed effects modelling with horse modelled as a random effect and time, treatment, and their interaction modelled as fixed effects. A Shapiro-Wilk test was used to test for normalcy and data were log_10_-transformed as appropriate to meet statistical assumptions. Microscopic pathology scores were rank-transformed and analyzed using linear-mixed effects modelling. Post hoc testing of pairwise combinations of times within treatment and treatment within time were made using the method of Sidak. [45] Model fit was assessed using diagnostic plots of residuals. Data were analyzed using S-PLUS statistical software (Version 8.2, TIBCO Inc., Seattle, WA) unless otherwise noted. Significance was set at P < 0.05 for all analyses.

## Results

### Non-infected wounds

**Fig 1:**
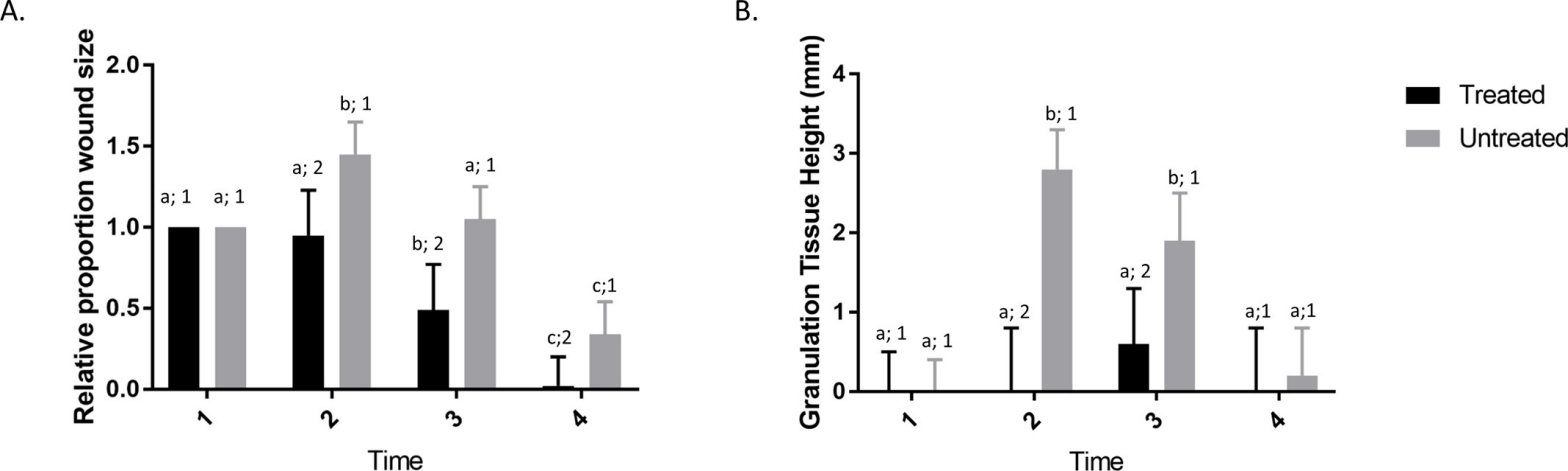
GaM treatment speeds wound closure and reduces formation of exuberant granulation tissue in non-infected wounds. A) Proportion of wound size relative to time 1 for both control and treated wounds. B) Granulation tissue height at each time point for both treated and untreated wounds. Bars represent means and error bars represent 95% CI. Columns with different letters were significantly different over time within each treatment group and columns with different numbers differed significantly between treatment groups within each time point (1 horse, 4 treated and 4 untreated wounds).

Initially, wound size was assessed in non-infected wounds in order to determine whether topical GaM exerted a beneficial effect on equine wound healing. There were significant effects of time, treatment, and their interaction. For the control wounds, the relative area was significantly lower at time 4 than all other time points. For the GaM wounds, times 3 and 4 were significantly lower than times 1 and 2. Effect of treatment was significant at times 2, 3, and 4, where GaM treated wounds were significantly smaller than controls (Fig 1A). In addition to wound size, wound height was also assessed as an indicator of exuberant granulation tissue. There were significant effects of treatment that depended on time. At time 1, there were no differences between treatments. Wounds were significantly higher in untreated than treated wounds at times 2 and 3, but not on time 4. While granulation height increased significantly at time 2 and 3 for control wounds, the GaM-treated wounds remained flat (Fig 1B).

These data suggest GaM exerted significant beneficial effects on wound size and prevention of exuberant granulation tissue formation in non-infected equine wounds. In order to gain insight into the possible mechanism(s) by which GaM exerted these effects, expression of genes known to be important in equine wound healing was assessed at time 4. [46] Specifically, we examined expression of the following genes: *IL-10*, *IL-1β*, *IL-2*, *IFN-γ*, *CXCL1*, *PDGF-A*, *VEGF-A*, *TGF-β*, *MMP1*, *MMP7*, *MMP9*, Serpine, *TIMP1*, *COL1A1*, *COL14A1*, and vitronectin. Expression of *IL-2*, *IFN-γ*, *TGF-β*, and vitronectin were significantly (P < 0.05) greater in treated than untreated wounds, whereas expression of *CXCL1*, *MMP1*, and *MMP9* were significantly greater in untreated wounds (Table 2). Taken together, these data indicate that uninfected GaM-treated wounds heal faster, with less exuberant granulation in wounds than uninfected wounds that were not treated with GaM. Moreover, gene expression data suggest that GaM exerted this beneficial effect by reducing inflammation, promoting expression of genes associated with healing, and reducing inflammation.

**Table 2:** Gene expression altered by GaM treatment at time 4 in non-infected equine wounds.

Gene	Treated—Mean (95% CI)	Untreated—Mean (95% CI)	P-value
*IL-10*	5.6 (3.3 to 9.5)	2.7 (1.3 to 5.7)	0.0986
*IL-1b*	3.8 (1.3 to 11.3)	15.2 (3.3 to 70.1)	0.1278
***IL-2***	**3.2 (1.8 to 5.9)**	**0.7 (0.3 to 1.5)**	**0.0108**
*IFN-γ*	**3.8 (2.7 to 4.9)**	**0.7 (-0.8 to 2.3)**	**0.0082**
***CXCL1***	**3.1 (1.4 to 7.0)**	**13.6 (4.3 to 42.8)**	**0.045**
*PDGFA*	1.1 (0.8 to 1.4)	0.7 (0.2 to 1.1)	0.0888
*VEGFA*	0.7 (0.5 to 0.8)	0.5 (0.3 to 0.7)	0.156
***TGF-β***	**4.3 (3.2 to 5.3)**	**1.6 (0.1 to 3.0)**	**0.0114**
***MMP1***	**43.0 (18.6 to 99.6)**	**596.9 (181.8 to 1,959.6)**	**0.0049**
*MMP7*	0.4 (0.2 to 0.8)	0.2 (0.1 to 0.4)	0.07
***MMP9***	**10.0 (-2.3 to 22.3)**	**38.9 (21.5 to 56.3)**	**0.0172**
Serpine	3.7 (2.3 to 5.0)	3.0 (1.1 to 4.8)	0.491
*TIMP1*	1.7 (0.0 to 3.5)	4.2 (1.7 to 6.6)	0.0991
*COL1A1*	12.4 (7.7 to 17.0)	4.2 (-2.4 to 10.9)	0.0528
*COL14A1*	1.7 (0.9 to 2.5)	1.5 (0.4 to 2.6)	0.7731
**Vitronectin**	**4.6 (3.4 to 5.8)**	**1.5 (-0.2 to 3.2)**	**0.0122**

Gene expression (arbitrary units) at Time 4 (treatment day 21) of GaM-treated and untreated wounds (1 horse, 4 treated and 4 untreated wounds). Gene expression is normalized to GAPDH and data are expressed as mean (95% CI) relative to normal skin. Genes whose expression differs significantly between treated and untreated wounds are bolded.

### Infected wounds

**Fig 2:**
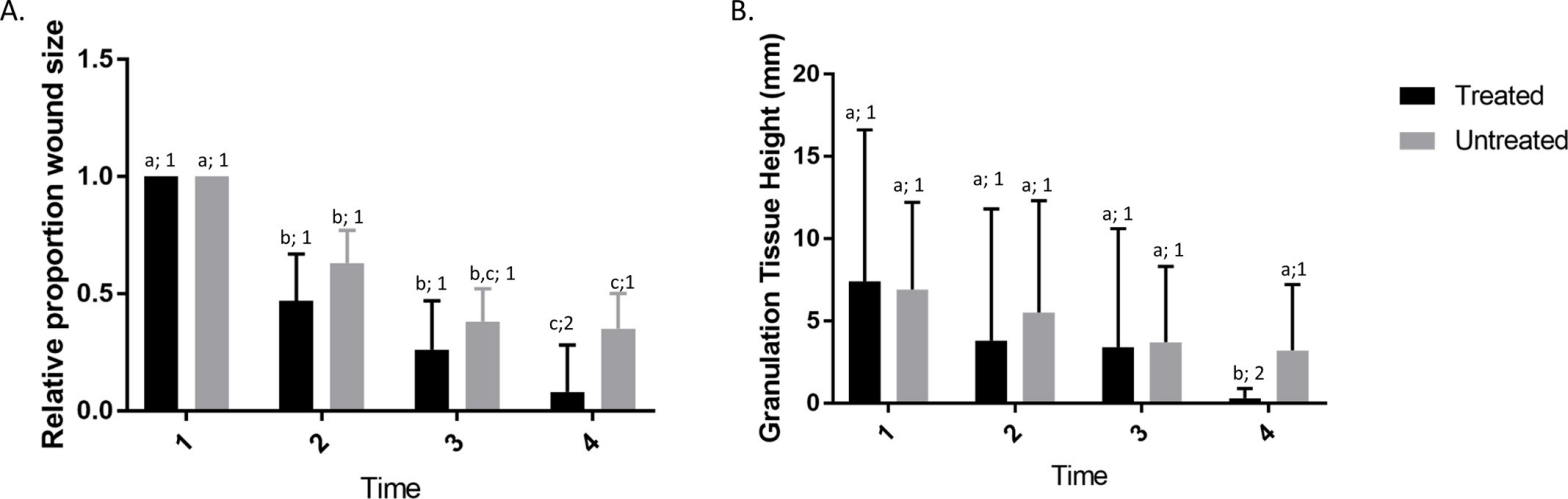
GaM treatment speeds wound closure and reduces formation of exuberant granulation tissue in infected wounds. A) Proportion of wound size relative to time 1 for both control and treated wounds. B) Granulation tissue height at each time point for both treated and untreated wounds. Bars represent means and error bars represent 95% CI. Columns with different letters were significantly different over time within each treatment group and columns with different numbers differed significantly between treatment groups within each time point (5 horses, 20 treated and 20 untreated wounds).

The vast majority of equine wounds are infected due to the manner by which horses sustain wounds and the environments in which horses are kept. Therefore, it was important that we examine the effects of GaM on infected equine wounds. We examined the effects of GaM on chronic equine wounds infected with *S*. *aureus*, a common pathogen of naturally occurring equine wounds. Significant effects of time, treatment, and their interaction (Fig 2) were noted in infected wounds. For the control wounds, the relative wound area was significantly lower than time 1 for all other times, and values were significantly lower than time 2 at times 3 and 4, but time 3 and time 4 did not differ significantly. For the GaM wounds, values were significantly lower than time 1 for all other weeks, and time 4 was significantly lower than time 2 and 3 but no other differences were significant. Effects of treatment within time were only significant at time 4 when GaM treated wounds were significantly smaller than controls (Fig 2A). As with non-infected wounds, wound height was also assessed as an indicator of exuberant granulation tissue. There was significant interaction of effects of time and treatment. For the control wounds, there were no significant effects of time (P > 0.05). For the GaM wounds, although values tended to decreased this difference was only significant at time 4, when values were significantly (P < 0.05) lower than all other times, and also were significantly (P < 0.05) lower than controls (Fig 2). Taken together, these data suggest the topical GaM exerts similar phenotypic effects on both infected and non-infected equine wounds.

### Effect of GaM on bacterial bioburden

**Fig 3:**
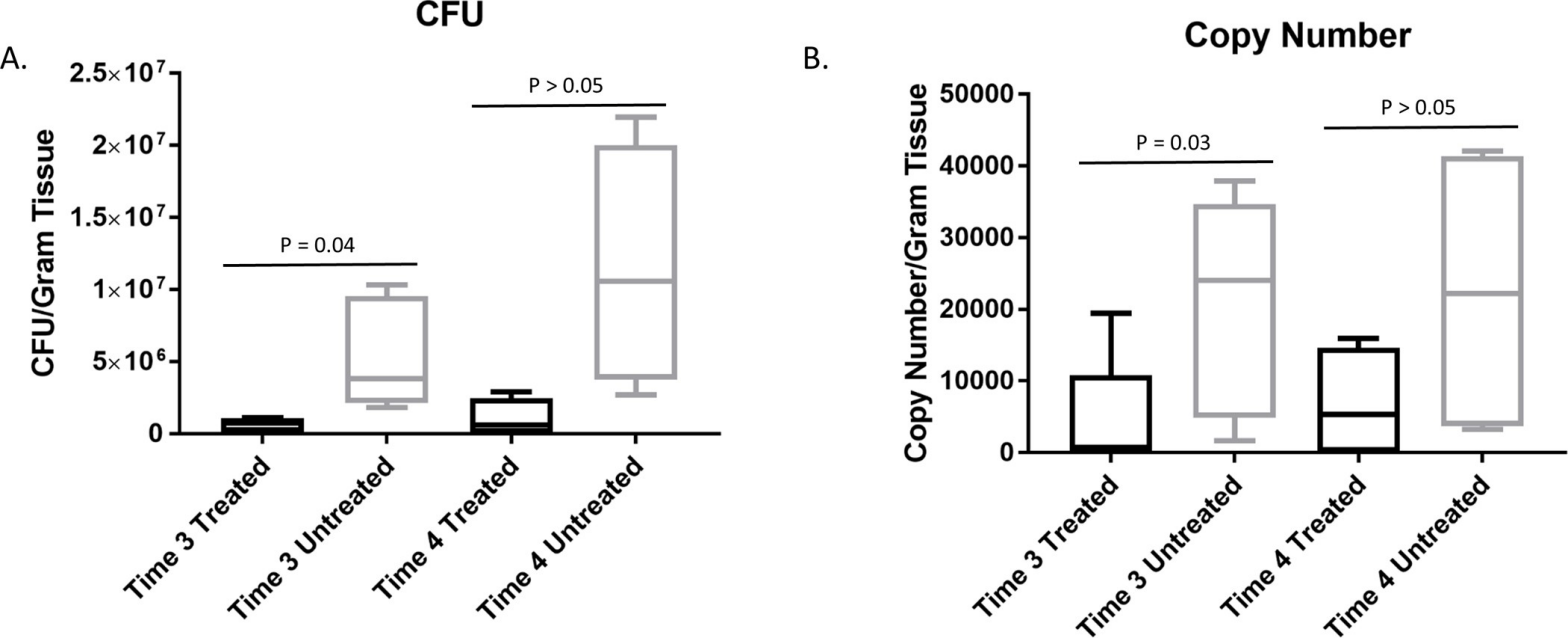
GaM treatment reduces wound bioburden of *S*. *aureus*. A) CFU of *S*. *aureus* per gram of tissue as determined by quantitative culture of wound biopsies and times 3 and 4. B) Copy number of *S*. *aureus* per gram of tissue as determined by real time qPCR from tissue biopsies. Box and whisker plots where horizontal bar represents median value, box represents the 25^th^ to 75^th^ percentiles and whiskers represent 5^th^ to 95^th^ percentiles. Columns with different letters were significantly different over time within each treatment group and columns with different numbers differed significantly between treatment groups within each time point (5 horses, 20 treated and 20 untreated wounds).

We next examined the effects of GaM on bacterial concentrations in these wounds. Bacterial concentrations were examined at times 3 and 4 because these were the time points when the greatest phenotypic differences existed for the wounds. Bacterial concentrations were quantified using both *S*. *aureus* selective quantitative culture and qPCR with *S*. *aureus*-specific primers to ensure the most robust results. The mean number of CFU per gram of tissue were significantly (P = 0.04) lower for the treated wounds (mean, 284,592; 95% CI, 46,886 to 1,727,456) than for the untreated wounds (mean, 4,329,463; 95% CI, 337,945 to 55,465,509) at time 3 (Fig 3A). There were no significant differences at time 4. Similarly, the copy numbers of *S*. *aureus* were significantly (P = 0.03) lower for the treated wounds (mean, 1,111; 95% CI, 154 to 8,016) than the untreated wounds (13,145; 95% CI, 1,594 to 40,994) at time 3. There were no significant differences in copy numbers at time 4 (Fig 3B).

### Effect of GaM on histology scores and gene expression in infected wounds

**Fig 4:**
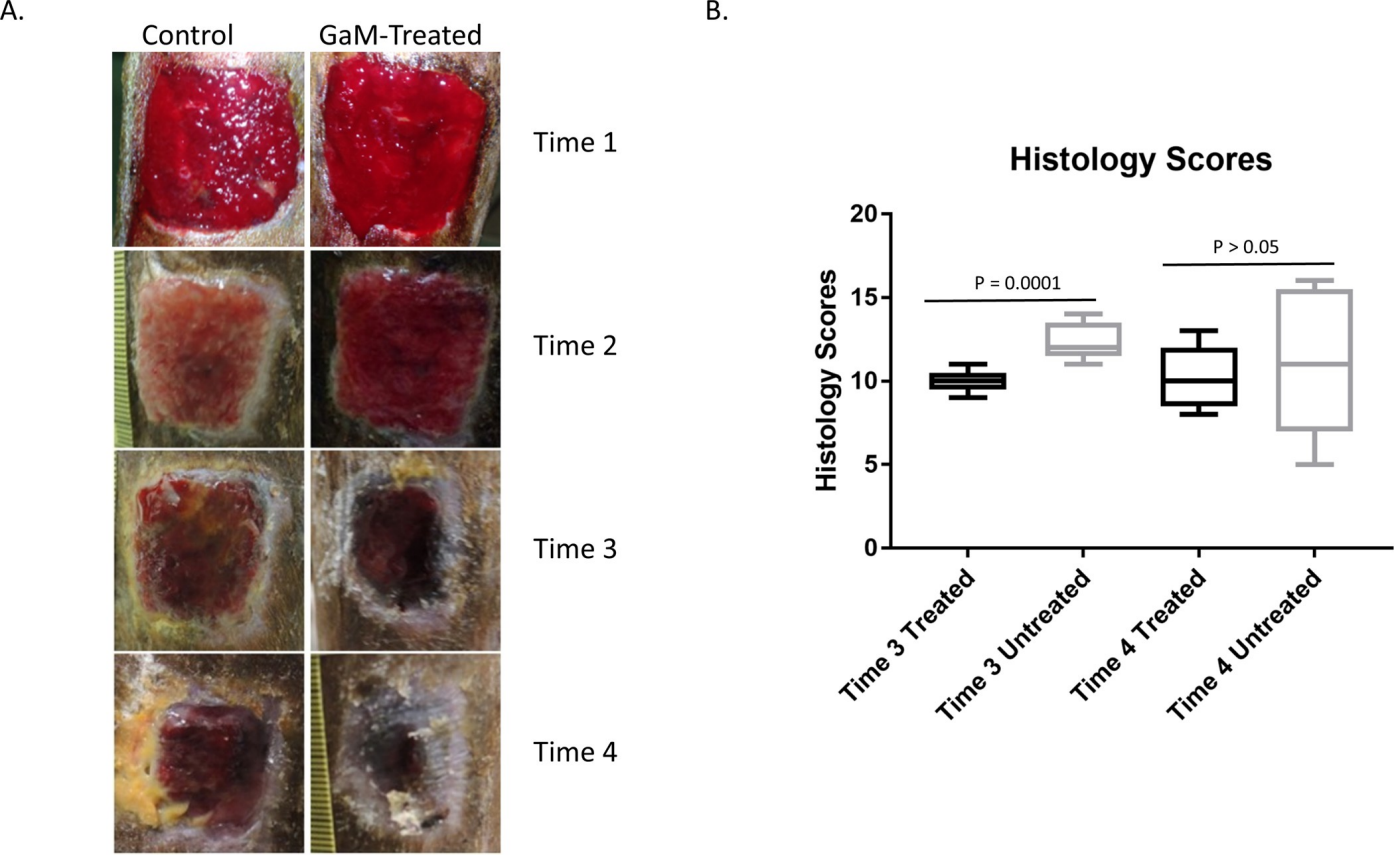
GaM improves gross appearance and microscopic pathology scores of infected wounds. A) Representative images of treated and untreated wounds at each time point. B) Summed histology scores from biopsies obtained at times 3 and 4.

**Fig 5:**
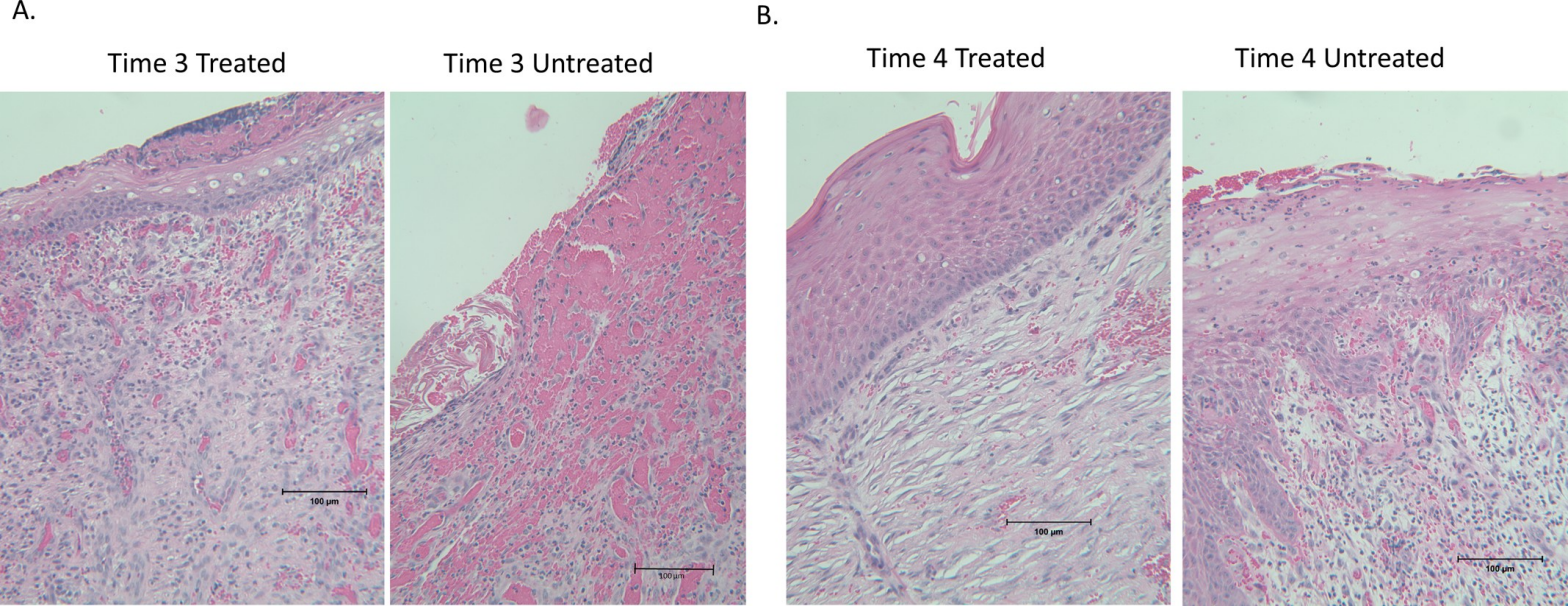
Representative histology images. H&E stained slides from biopsies acquired at times 3 and 4.

We also examined microscopic pathology scores at both times 3 and 4. The histology grade data were rank-transformed and analyzed using linear-mixed effects modeling. The mean rank of histology scores was significantly (P = 0.0001) lower for the treated limbs (mean rank, 3.1; 95% CI; 1.0 to 5.2) than for the untreated limbs (mean rank, 4.8; 95% CI, 4.0 to 5.6) at time 3. As with bacterial concentrations, there was no significant difference (P = 0.64) between treatments in the ranks of the histology grades for the treated group (mean, 5; 95% CI, 1 to 9) and the untreated wounds (mean, 6; 95% CI, 0.5 to 11.5) at time 4 (Fig 4B). Representative images depicting the gross appearance of the wounds and representative histology images are shown in Figs 4A and 5 respectively. Expression of the same genes examined in the non-infected wounds was examined in infected wounds at both times 3 and 4. At time 3, expression did not differ significantly for any gene except *IFNγ*, which was marginally higher in treated than untreated wounds (Table 3). At time 4, expression did not differ significantly for any gene except *TGFβ* and serpine (Table 3).

**Table 3:** Gene expression altered by GaM treatment at times 3 and 4 in infected equine wounds.

	Time 3			Time 4		
Gene	Treated	Untreated	P-value	Treated	Untreated	P-value
*IL-10*	2.0 (0.2 to 19.5)	2.4 (0.3 to 20.5)	0.80	1.9 (0.1 to 27.6)	0.9 (0.3 to 3.3)	0.16
*IL-1β*	2.2 (0.2 to 22.7)	2.7 (0.2 to 33.6)	0.79	2.1 (0.1 to 36.3)	1.1 (0.2 to 6.1)	0.31
*IL-2*	2.4 (0.5 to 11.2)	1.5 (0.5 to 4.8)	0.29	2.6 (0.7 to 4.6)	1.2 (-1.0 to 3.3)	0.11
*IFN-γ*	1.4 (0.8 to 2.5)	0.9 (0.6 to 1.4)	**0.04**	1.5 (0.4 to 2.7)	1.2 (0.4 to 2.0)	0.33
*CXCL1*	3.6 (0.5 to 25.8)	4.0 (0.6 to 28.2)	0.85	6.0 (-0.4 to 12.4)	3.9 (-5.1 to 12.9)	0.51
*PDGFA*	0.8 (0.0 to 1.6)	1.0 (0.1 to 1.9)	0.47	0.8 (0.4 to 1.6)	0.6 (0.2 to 1.6)	0.41
*VEGFA*	0.5 (0.4 to 0.7)	0.6 (0.4 to 1.0)	0.28	0.7 (0.2 to 1.1)	0.7 (0.1 to 1.2)	0.98
*TGF-β*	1.6 (0.6 to 4.4)	1.4 (0.5 to 3.9)	0.75	1.9 (0.8 to 4.7)	1.0 (0.6 to 1.8)	**0.04**
*MMP1*	70.5 (-140.2 to 281.2)	237.8 (17.3 to 458.3)	0.09	56.0 (-82.6 to 194.6)	147.6 (-19.4 to 314.6)	0.18
*MMP7*	1.0 (0.2 to 5.6)	1.1 (0.2 to 5.3)	0.95	1.8 (-0.2 to 3.7)	0.6 (-1.3 to 2.5)	0.15
*MMP9*	4.6 (0.9 to 25.1)	10.4 (2.8 to 38.0)	0.14	5.0 (-2.4 to 12.4)	7.4 (-3.1 to 17.8)	0.52
Serpine	2.9 (0.7 to 12.5)	3.0 (0.7 to 13.8)	0.94	3.8 (1.8 to 5.9)	2.2 (1.0 to 3.4)	**0.02**
*TIMP1*	1.6 (-0.7 to 3.8)	2.8 (1.0 to 4.6)	0.12	1.7 (-0.1 to 3.5)	2.0 (0.4 to 3.5)	0.60
*COL1A1*	8.9 (3.3 to 24.2)	8.6 (2.6 to 28.6)	0.93	8.9 (-13.2 to 20.2)	9.7 (-4.3 to 23.8)	0.87
*COL14A1*	0.5 (0.1 to 0.9)	0.3 (-0.1 to 0.8)	0.29	1.0 (0.2 to 1.7)	0.6 (-0.4 to 1.6)	0.33
Vitronectin	3.5 (0.8 to 15.2)	2.7 (0.7 to 10.8)	0.61	3.9 (0.8 to 7.1)	1.6 (-1,3 to 4.6)	0.09

Gene expression, mean arbitrary units and 95% CI, of select genes examined at times 3 and 4. Bolded and underlined P-values are significant (P < 0.05).

## Discussion

The use of 0.5% GaM as a topical wound treatment resulted in more rapid reduction in wound size, reduced formation of exuberant granulation tissue, reduced *S*. *aureus* bioburden, and improved wound morphology histologically in equine wounds. Given the frequency with which horses incur wounds, inherent difficulties associated with wound healing on the distal limb of horses, and the increasing identification of multi-drug resistant (MDR) bacteria associated with equine wounds, an inexpensive and effective wound care product that exerts both host effects and antimicrobial effects holds great promise.

Gallium has been studied extensively as an antimicrobial agent. [16–26, 47] The antimicrobial properties of gallium have been attributed to its similarities to ferric iron (Fe^3+^). These chemical similarities result in uptake by bacteria which ultimately causes bacterial death because gallium cannot be reduced at physiological pH values for metabolic processes, whereas ferric iron can be reduced to ferrous iron. [22] Previous studies have demonstrated that GaM has antimicrobial properties against several types of bacteria including *S*. *aureus* and MRSA. [16, 48] Here, we have demonstrated that GaM possesses antimicrobial activity against *S*. *aureus in vivo*. Presumably, this reduction in bioburden contributed to the beneficial effects of GaM on wound healing. This reduction in bacterial load may also have played a role in the reduced inflammatory morphology seen on histopathology, and greater reduction in wound size in treated vs. untreated wounds. Delayed wound healing or cessation of wound healing is typically multifactorial, but one of the most common causes of delayed healing is contamination/infection of the wound. [49] Infection, defined as a bacterial burden of 10^5^ CFU per gram of tissue, delays wound healing due to a release of damaging free radicals, increased oxygen consumption, and endotoxin production which can result in thrombosis of wound microvasculature, as well as catalyzing the release of more inflammatory mediators. [50] Ultimately, these events lead to chronic inflammation and delays wound healing. The antimicrobial effects of gallium demonstrated in the current study may explain the improvements in wound healing and decreased inflammation noted in treated wounds compared to untreated wounds. This supports the use of gallium in the topical treatment of contaminated wounds of the distal limb of horses.

The fact that GaM exerted beneficial effects in non-infected wounds indicates that antimicrobial effects of GaM alone do not explain the mechanism by which wound healing was improved, and suggests the possibility of host-directed effects. Previous studies have shown similar effects, with the use of topical gallium nitrate in a porcine model resulting in improved wound healing potentially by repression of matrix metalloproteinase enzyme activity in the wound, and increase in matrix synthesis and acceleration of keratinocyte motility. [26, 51] Unregulated expression of the MMP family of enzymes beyond the initial inflammatory stages of wound healing has been associated with delayed wound healing in both people and horses. [46] Interestingly, we also observed decreased expression of both MMP1 and MMP9 after treatment of non-infected wounds with GaM, further indicating that GaM inhibits this family of enzymes *in vivo*. We did not, however, observe this same reduction of MMPs in infected wounds treated with GaM at the same time-point. The reasons for this discrepancy are unclear. Chronically infected wounds have increased MMP expression and it is possible that the concentration of GaM utilized in this study was insufficient to inhibit the increased expression of these enzymes. [52] In addition, there was a great deal of variation in MMP expression in the infected wounds and, while MMP expression tended to decrease, we lacked adequate power to detect a significant difference of the magnitude observed in our small sample of horses because of the high variability of MMP gene expression.

We consistently observed increased expression of transforming growth factor-β (TGF-β) in both non-infected and infected wounds. TGF-β1 has been shown to promote more efficient acute inflammation, and progression of wound healing. [53, 54] This acute inflammatory response is critical to wound healing and horses have been shown to have a weak initial inflammatory response in wounds of the distal limb which contributes to the delayed healing observed in this location of horses. [53] Importantly, differences in TGF-β have been linked to these differences in the acute inflammatory response between horses and ponies. [54] The consistently observed increase in TGF-β in wounds treated with GaM suggest a mechanism by which GaM resulted in improved wound healing above GaM’s antimicrobial properties.

Interestingly, we also consistently observed significantly increased interferon-γ (IFN-γ) expression in response to GaM treatment. IFN-γ, similar to TGF-β, has been shown to stimulate the inflammatory phase of wound repair and improves phagocyte mediated activity against *S*. *aureus*. [55] In addition, there is evidence of cross-talk between TGF-β and IFN-γ, but IFN-γ reportedly delays wound healing, antagonizes the beneficial effects of TGF-β, and is associated with impaired wound healing in people and other animal models. [56, 57] The role of IFN-γ specific to equine wound healing has not been extensively studied but, as with other species, the balance between pro-inflammatory IFN-γ and other cytokines is important and wound specific. The mild increase in IFN-γ observed in this study in concert with improved wound healing may represent improved phagocyte function and restoration of the critically important early and robust inflammatory phase lacking in wounds of the equine distal limb.

Wounds of the equine distal limb are prone to the formation of exuberant granulation tissue. This is characterized by increased cellular proliferation, delayed wound contraction, and delayed epithelialization. [58–60] Development of exuberant granulation tissue is due to many factors including weak but prolonged inflammation, infection, motion, and other factors. Mechanistically, it is thought that delayed transformation of fibroblasts to myofibroblasts results in continued proliferation of fibroblasts and decreased wound contraction and extracellular matrix (ECM) production. [61–63] The early, insufficient, acute inflammatory response that ultimately results in persistent, chronic inflammation has been associated with lower expression of TGF-β1 in horses, compared to the more efficient initial inflammatory response in ponies. [54] Thus, the GaM-induced reduction in exuberant granulation tissue might be attributable to increased TGF-β expression. This same effect might have reduced wound size because exuberant granulation tissue, which is raised above the wound surface, contracts poorly due to fibrosis, and serves as a physical barrier to epithelialization. The antimicrobial effects of GaM might also have contributed to reduced exuberant granulation tissue because it is more likely to form in wounds with contamination and chronic infection.

This continuum of beneficial effects (*i*.*e*., increased TGF-β, decreased MMPs, decreased exuberant granulation tissue, and decreased bioburden) collectively lead to improved wound healing as evidenced by wound size over time and microscopic pathology scores. Untreated wounds had protracted inflammation, decreased re-epithelialization, and slowed contraction, as well as increased fibroplasia. This is similar to previous findings, where gallium has been shown to induce beneficial effects on wound healing. [51] Gallium maltolate decreased histology scores in treated wounds, and decreased the size and tissue height of wounds. These findings indicate that further clinical evaluation of GaM as an adjunctive treatment to reduce formation of excessive granulation tissue and to prevent development of a chronic inflammatory response in equine wounds is warranted.

There were several important limitations to this study. First, the sample size was relatively small, and this limitation was further compounded by the extent of inter-individual variation in responses of the horses to wound healing and treatment. This reduced our ability to detect significant differences in some parameters between treated and untreated wounds. A specific example where this may have occurred is the bioburden data at time 4 where the small sample size and individual variation prevented detecting significant differences although the pattern appears to demonstrate substantial, although not significant, differences related to treatment. Similarly, only one horse (8 wounds) was used for the non-infected model which is less than ideal due to lack of independence of samples. The rationale for this decision was based on the lack of clinical recapitulation with this model as non-infected, open wounds are not a clinical entity in equine veterinary medicine. This decision was also based on a desire to use as few animals as possible, a practice in-line with recommendations and guidelines from governing bodies overseeing research involving use of animals. Also in this model, non-infected wounds were only assumed to be non-infected based on clinical appearance of the wounds (*i*.*e*., cultures were not performed). It is possible that subclinical infection existed and, if true, suggests that the primary benefit of GaM lies in its antimicrobial properties and not host-directed effects. Another limitation is the fact that GaM was delivered in a petroleum medium, which has been associated with increased bacterial growth and formation of exuberant granulation tissue in horses. [64] Finally, we used a model of equine wound healing which has several important advantages for research; however, it is unclear if the same beneficial effects of GaM would be observed in naturally occurring equine wounds. The effectiveness of this treatment in the equine distal limb model warrants further clinical trials in non-healing wounds infected with other infectious agents (*Pseudomonas aeruginosa*), and with MDR bacteria *in vivo*, as well as with chronic, non-infected wounds.

Despite these limitations, we demonstrated GaM treatment of distal limb wounds of horses with exuberant granulation tissue resulted in a significant reduction in wound size and granulation tissue height, and improved wound morphology compared to control wounds, when applied between weeks 2 and 4 following creation of a full-thickness skin wound. GaM applied topically to wounds had a significant anti-microbial effect, which likely also played a role in the improved wound healing. Based on the results of this study, GaM could be a useful adjunctive therapy in the management of wounds of the distal limb of horses that are infected and that are prone to develop exuberant granulation tissue. Further studies are warranted to examine its effect against MDR organisms, antimicrobial effects in other treatment applications, and translational studies.

## Supporting information

S1 Fig Schematic and image of equine wound model used in this study. (TIF)

S2 Fig Schematic demonstrating how granulation height was measured. (TIF)

S1 Checklist The ARRIVE guidelines checklist. (DOCX)
