# Supplementary figures and images for: Effect of gallium maltolate on a model of chronic, infected equine distal limb wounds
Source: PLoS One. 2020 Jun 19;15(6):e0235006. doi: 10.1371/journal.pone.0235006 (PMC7304909; doi:10.1371/journal.pone.0235006)

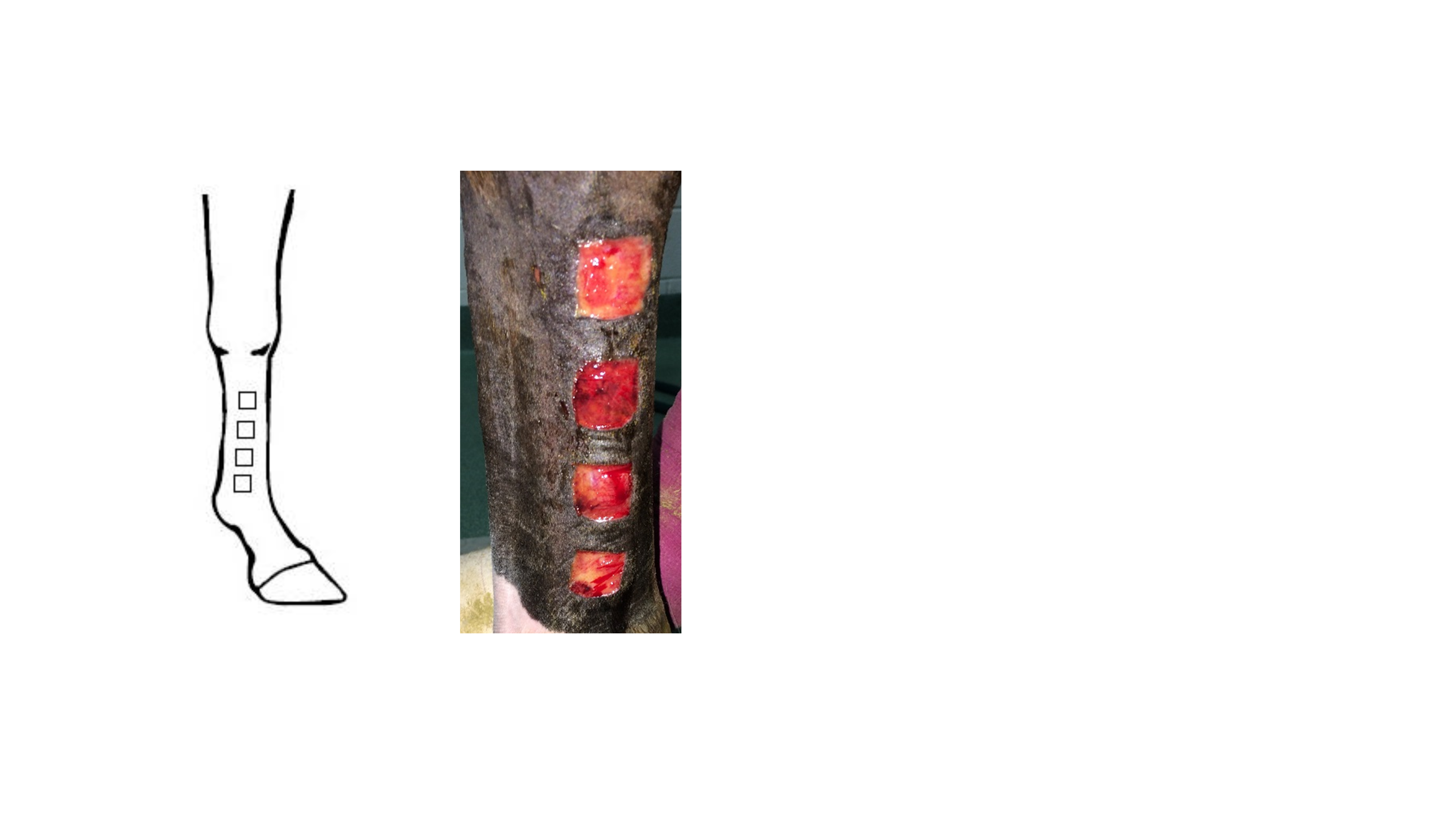

Supplement: S1 Fig — (TIF) [file pone.0235006.s001.tif]

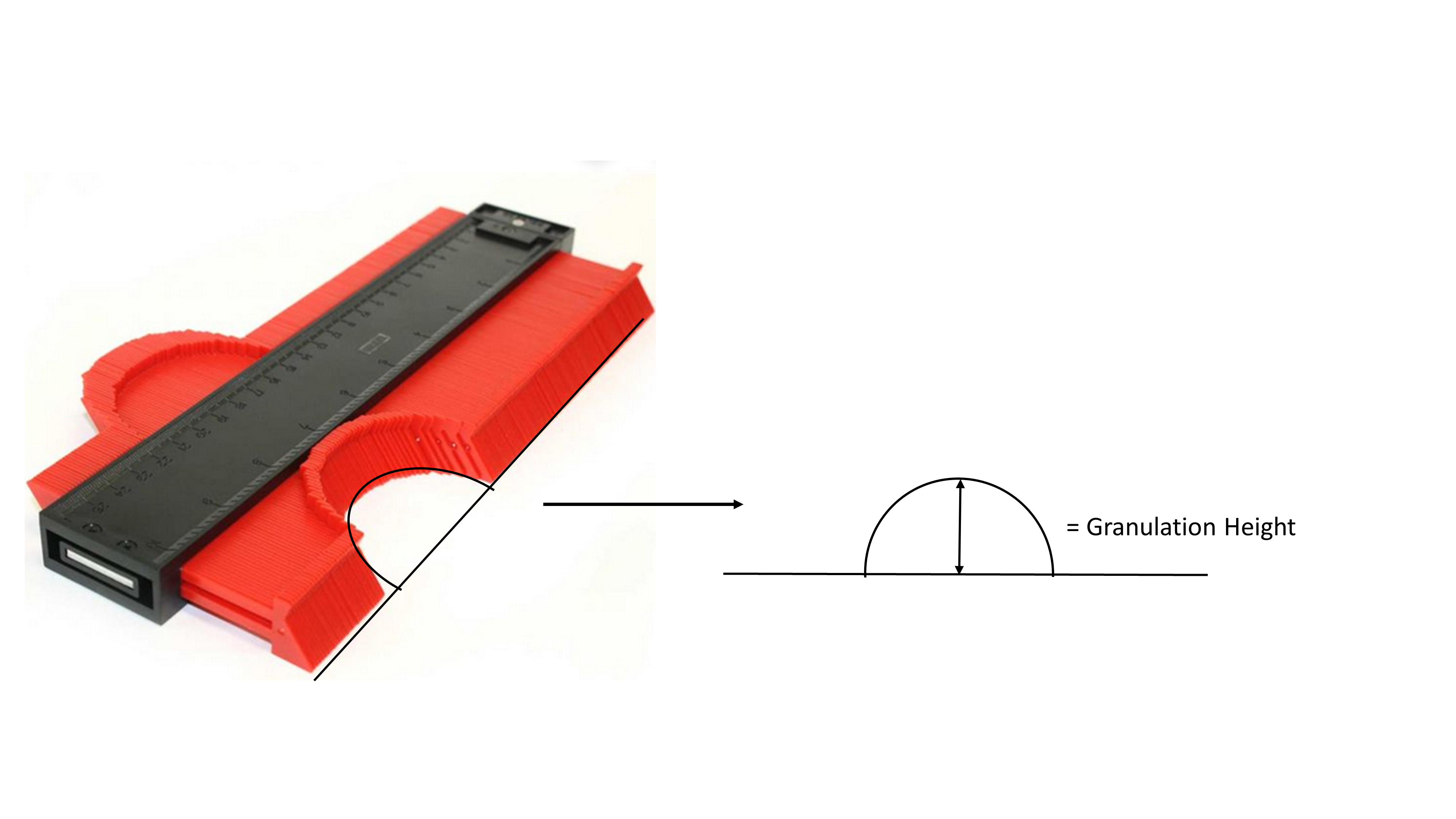

Supplement: S2 Fig — (TIF) [file pone.0235006.s002.tif]
