# Supplementary material for: Effect of gallium maltolate on a model of chronic, infected equine distal limb wounds
Source: PLoS One. 2020 Jun 19;15(6):e0235006. doi: 10.1371/journal.pone.0235006 (PMC7304909; doi:10.1371/journal.pone.0235006)

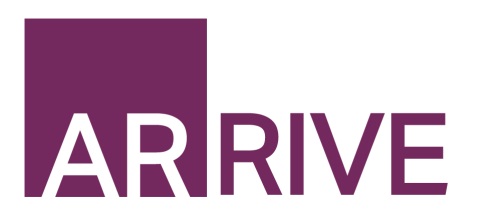


The ARRIVE Guidelines Checklist

Animal Research: Reporting In Vivo Experiments

Carol Kilkenny^1^, William J Browne^2^, Innes C Cuthill^3^, Michael Emerson^4^ and Douglas G Altman^5^

*^1^The National Centre for the Replacement, Refinement and Reduction of Animals in Research, London, UK, ^2^School of Veterinary Science, University of Bristol, Bristol, UK, ^3^School of Biological Sciences, University of Bristol, Bristol, UK, ^4^National Heart and Lung Institute, Imperial College London, UK, ^5^Centre for Statistics in Medicine, University of Oxford, Oxford, UK.*

|  | | ITEM | RECOMMENDATION | Section/ Paragraph |
| --- | --- | --- | --- | --- |
| 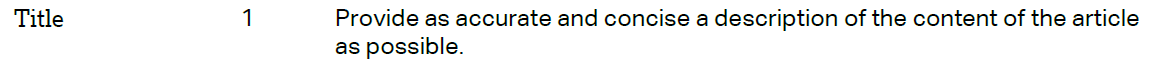 | | | Title |  |
| 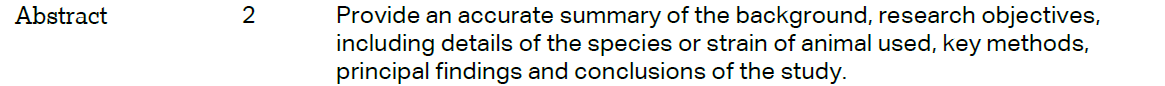 | | | Abstract |  |
| INTRODUCTION | | |  |  |
| 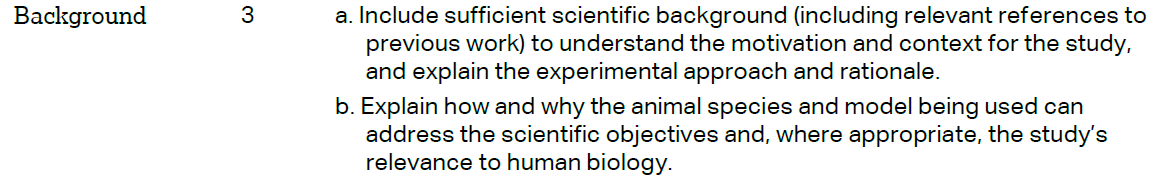 | | | Paragraphs 1 & 2 |  |
| 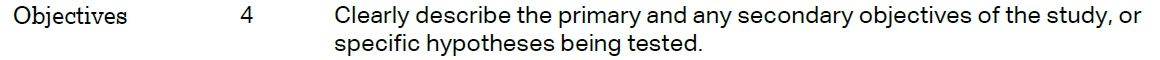 | | | End of Paragraph 2 |  |
| METHODS | | |  |  |
| 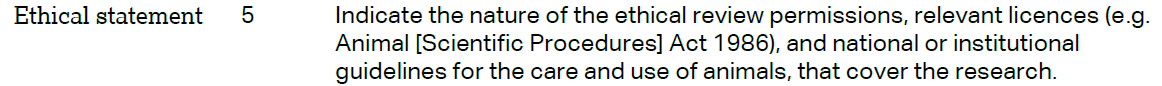 | | | M and M 1^st^ paragraph |  |
| 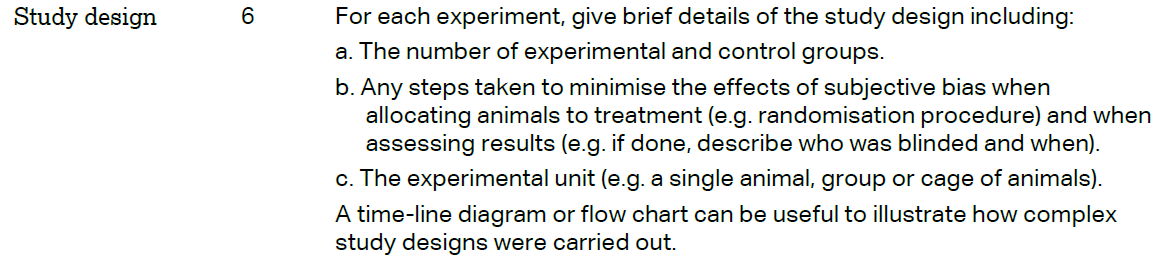 | | | M and M paragraphs  1, 2, 4, 5 |  |
| 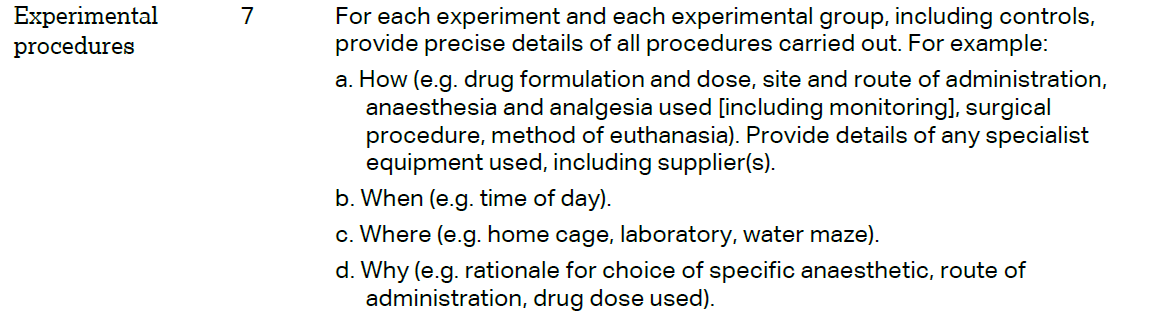 | | | M and M paragraphs  2 & 3 |  |
| 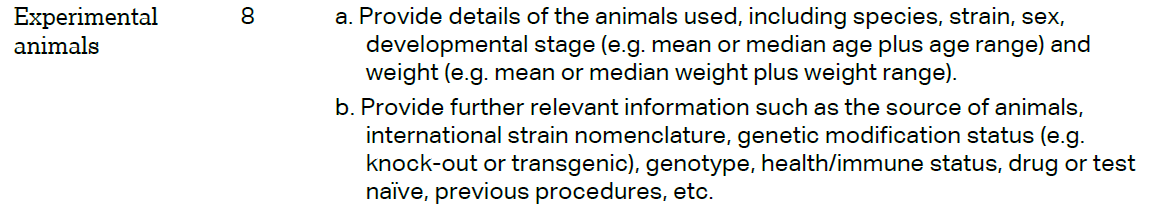 | | | M and M paragraph 1 |  |

The ARRIVE guidelines. Originally published in *PLoS Biology*, June 2010^1^

| 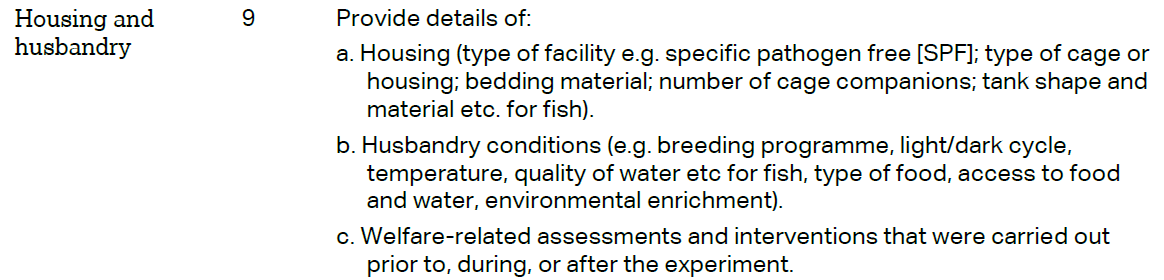 | M and M paragraph 1 | |
| --- | --- | --- |
| 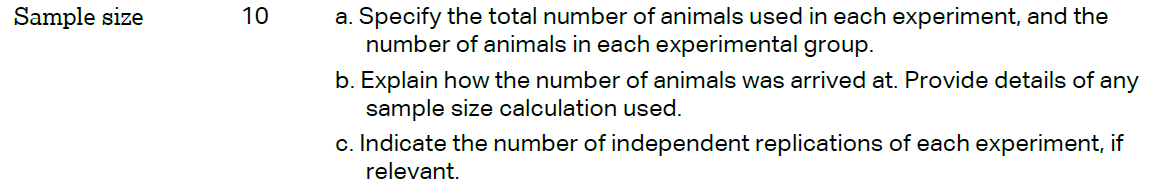 | M and M paragraph 1 | |
| 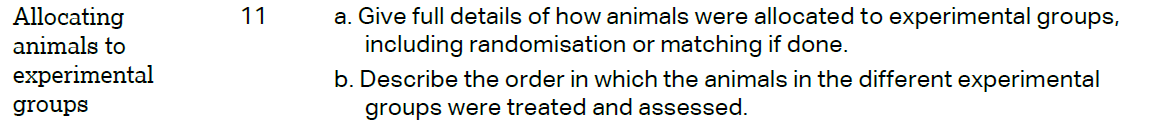 | M and M paragraph 1 | |
| 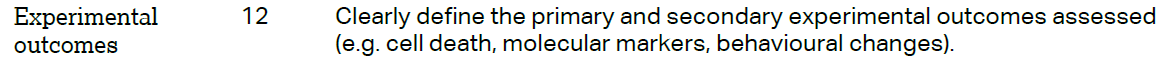 | M and M paragraphs 3,4,5,6 | |
| 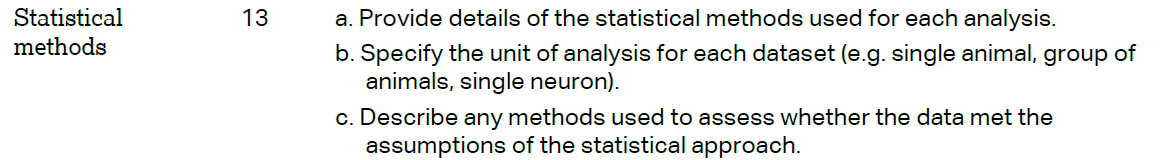 | M and M paragraph 7 | |
| RESULTS |  | |
| 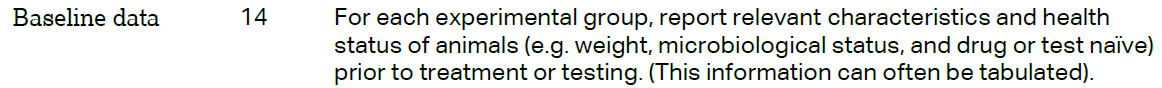 | M and M paragraph 1 | |
| 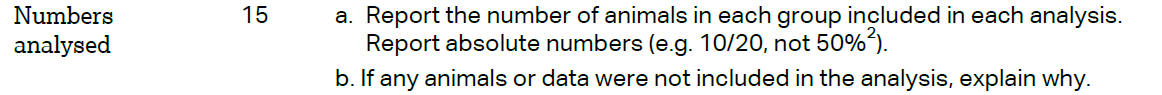 | M and M paragraph 1 | |
| 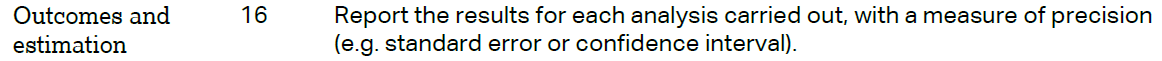 | Tables 2,3 Figures 1,2,3,4 | |
| 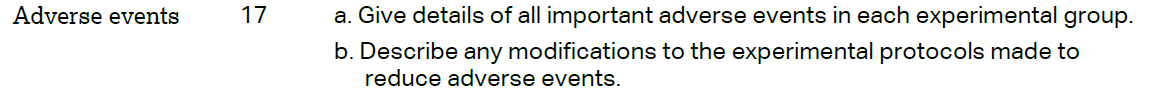 | N/A | |
| DISCUSSION |  | |
| 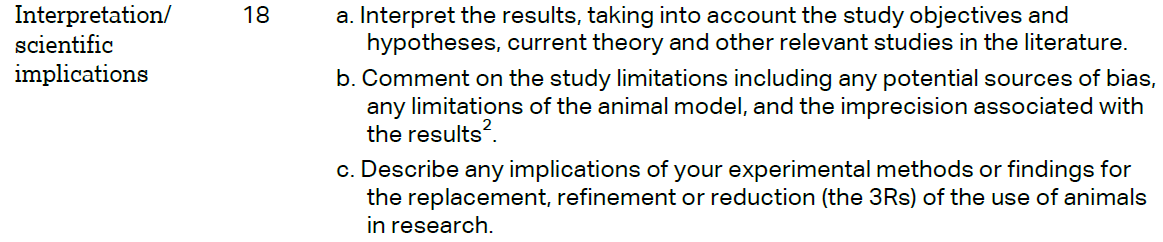 | Discussion paragraphs 1,3,4,5  Discussion paragraph 7 | |
| 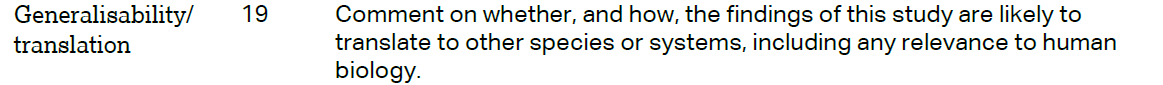 | Discussion paragraph 8 | |
| 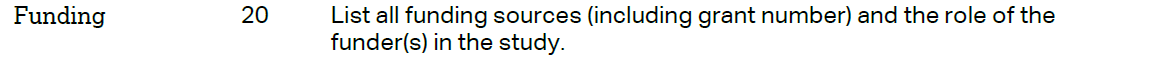 | | Acknowledgements |


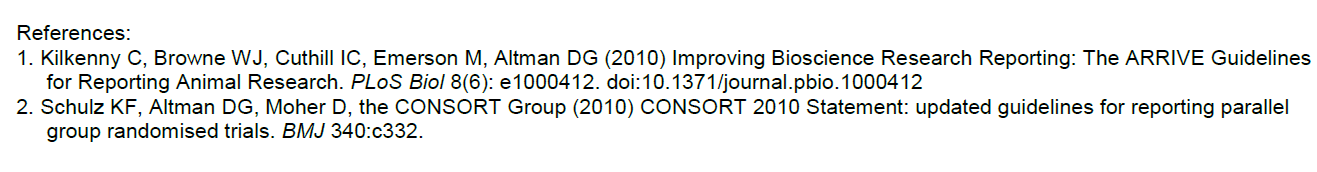

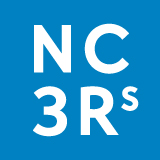

Supplement: S1 Checklist — (DOCX) [file pone.0235006.s003.docx]
